# The influence of contextual constraint and word length on eye movement control during Chinese reading

**DOI:** 10.3389/fpsyg.2025.1652627

**Published:** 2025-09-19

**Authors:** Weiqiong Jin, Yang Liu, Suiping Wang

**Affiliations:** ^1^School of Psychology, South China Normal University, Guangzhou, China; ^2^Philosophy and Social Science Laboratory of Reading and Development in Children and Adolescents (South China Normal University) Ministry of Education, Guangzhou, China; ^3^Guangzhou Baiyun Xingzhi Vocational and Technical School, Guangzhou, China

**Keywords:** contextual constraint, word length, eye movements, saccade target selection, Chinese reading

## Abstract

Given that Chinese text lacks explicit spaces to mark word boundaries, readers need to segment the continuous text into words of varying lengths. Contextual information helps determine word boundaries in Chinese reading. However, it remains unclear how contextual constraint and word length information guide eye movements during Chinese reading. To address this issue, the present study examined the relationship between contextual constraint and word length information in determining when and where to move the eyes in Chinese reading. We manipulated contextual constraint such that the target words were either predictable or unpredictable, and manipulated word length such that the target words were either single-character or three-character. The results demonstrated that both contextual constraint and word length influenced word skipping, fixation durations, saccade lengths, and landing positions. However, we did not find significant interactions between them across all measures. Moreover, Bayes factor analysis provided strong evidence for the absence of an interaction, suggesting that contextual constraint does not modulate the effect of word length on eye-movement control in Chinese reading. These findings advance our understanding of eye-movement control mechanisms in Chinese reading and provide empirical evidence for improving existing models of Chinese reading.

## Introduction

Reading is a fundamental and complex cognitive activity. During reading, readers need to dynamically adjust their eye movements to acquire and integrate textual information. A central question in eye movement control concerns the factors that determine both when and where the eyes move (Rayner, 1998, 2009). In alphabetic languages such as English, word length is one of the most important factors affecting eye movement control (Joseph et al., 2009; Plummer and Rayner, 2012; Rayner et al., 2011; White et al., 2005). Unlike English, Chinese text comprises continuous character strings without explicit spaces to mark word boundaries. Therefore, Chinese readers need to segment this continuous text into words of varying lengths during reading (Li and Pollatsek, 2020; Li et al., 2009). Previous studies have demonstrated that sentence context can facilitate word segmentation in Chinese reading (Huang and Li, 2020; Huang et al., 2021; Yang et al., 2012). Building on this, the present study examined how contextual constraint and word length information influence eye-movement behavior. Addressing this question will contribute to a deeper understanding of the mechanisms underlying Chinese reading.

In English reading, word length plays a role at the earliest and affects the entire process of word recognition. Word length information is easily acquired from the parafoveal vision, and it guides the eyes to the left of the word center, a position called the preferred viewing location (PVL) (Rayner, 1979). Moreover, word length also affects the word skipping and fixation times: short words are more likely to be skipped and receive shorter fixations than long words (Drieghe et al., 2004; Joseph et al., 2009; Plummer and Rayner, 2012; White et al., 2005).

However, the role of word length in reading Chinese is different from that in English. Specifically, word length effects are robust in the lexical recognition and post-lexical integration stages. For example, word length significantly influences fixation times and skipping rates: short words receive shorter fixations and are more frequently skipped than long words (Li et al., 2011; Li X.-W. et al., 2022; Ma et al., 2019; Zang et al., 2018). Nevertheless, it remains controversial whether word length guides saccade targeting, particularly whether readers use a word-based strategy to fixate near the word centers (Li et al., 2011; Ma et al., 2019; Yan et al., 2010). Early investigations found no evidence of a PVL in Chinese; instead, fixations distribute uniformly across characters (Yang and McConkie, 1999; Tsai and McConkie, 2003). However, Yan et al. (2010) reported different findings, showing that initial landing positions tended to land at the word center in single-fixation cases and at the word beginning in multiple-fixation cases. They proposed that saccade target selection is associated with parafoveal word segmentation: readers target the word center when segmentation is successful and the word beginning when it is not.

Subsequent studies have questioned this interpretation. Zang et al. (2013) artificially inserted spaces into Chinese texts to examine eye movement behavior under word-spaced and unspaced conditions. The results showed a similar pattern to Yan et al. (2010), as the difference in fixation distribution between single and multiple fixations still existed, regardless of the presence of visual cues marking word boundaries. Li and Shen (2013) further examined the joint effects of inserted spaces and word length on saccade targeting and found no tendency for readers to fixate at the word center, even when word boundaries were explicitly marked and word length varied. These findings suggest that word boundary and length information exert little influence on saccade targeting (also see Li et al., 2011; Ma et al., 2019).

Despite these findings suggesting that saccade targeting in Chinese is not word-based, these studies have limitations. First, they predominantly used low-constraint sentence contexts, which may encourage greater reliance on bottom-up processing (Balota et al., 1985; Dambacher, 2010; Rayner and Well, 1996). In Chinese reading, characters often serve as the basic processing units (Chen, 1996; Chen et al., 2003; Hoosain, 1991, 1992), potentially overshadowing the word length effect. Furthermore, artificially inserted word boundaries may disrupt the natural reading process and alter cognitive mechanisms (Bai et al., 2008). A recent study found that inserting spaces into Chinese text provides limited facilitative information (Huang et al., 2024). To more accurately assess word length effects under ecologically valid conditions, employing high-constraint contexts may strengthen readers’ ability to predict word boundaries, thereby clarifying the role of word length in natural reading.

A substantial body of research confirms that sentence context plays a critical role in reading (Reichle, 2021; Schwanenflugel and Shoben, 1985). Efficient reading relies on the ability to predict upcoming linguistic content using contextual cues (Kuperberg and Jaeger, 2016; Pickering and Gambi, 2018; Ryskin and Nieuwland, 2023; Wong et al., 2024). This predictive processing facilitates lexical access, as evidenced by eye-tracking studies: compared to low-constraint contexts, target words in high-constraint contexts receive shorter fixation durations and are more likely to be skipped (Ehrlich and Rayner, 1981; Rayner et al., 2005; Rayner and Well, 1996; Schotter et al., 2015). More importantly, due to the absence of explicit word boundaries, Chinese reading relies more on context and semantic processing to determine word boundaries (Huang and Li, 2020; Huang et al., 2021) and to guide saccade length (Liu et al., 2018). This increased reliance on contextual information may enhance the utilization of word length cues when they are available, particularly in high-constraint contexts. This raises a key question: how do contextual and word length information influence eye movement control in Chinese reading?

Exploring the relationship between contextual constraint and word length information is essential for testing and refining models of eye movement control in reading. The E-Z Reader model (Reichle et al., 2003) assumes serial word identification comprising two stages: a familiarity check (L1) and full lexical access (L2). In this model, contextual predictability primarily affects the L1 stage, while word length influences both L1 and L2. Given their operation at separate stages, the effects of context and word length are assumed to be additive rather than interactive (Rayner et al., 2011). In contrast, the SWIFT model (Engbert et al., 2002, 2005; Richter et al., 2006) posits parallel lexical processing of multiple words within the perceptual span, with processing speed and depth influenced by factors such as visual acuity, word frequency, and predictability. This framework allows for potential interactions among multiple variables. However, both models were primarily developed based on English reading and lack mechanisms for word segmentation, limiting their applicability to Chinese. The Chinese Reading Model (CRM; Li and Pollatsek, 2020; Li et al., 2009), based on an interactive activation architecture (McClelland and Rumelhart, 1981), incorporates word segmentation but does not explicitly address whether and how context and word length interact during lexical processing. This study directly examines whether contextual constraint and word length jointly influence eye movement control in Chinese reading through a rigorously controlled eye-tracking experiment. The results will advance our understanding of how information from various sources contributes to reading and provide empirical support for the development of a more comprehensive cognitive model of Chinese reading.

The current study aimed to examine the relationship between contextual constraint and word length information in determining when and where the eyes move during Chinese reading. We manipulated two variables—sentence context and word length. Sentence context was varied to make the target word high- or low- constraint. In the high-constraint context, the target word was highly predictable, whereas in the low-constraint context, it was unpredictable (see *Rating of materials*). In addition, target word length was manipulated such that the words were either single-character or three-character words. If contextual constraint and word length information jointly influence when and where the eyes move, interactive effects should occur—we expect contextual constraint and word length to interact in terms of fixation times, skipping rates, and landing positions of target words. For example, when the context makes a word highly predictable and a consistent word-length cue is present, the likelihood of fixating on or skipping the word may be greater than would be expected based on either information source alone. Conversely, another possibility is that word length and sentence context do not interact to influence fixation times and word skipping. In this case, although both sources of information may influence skipping or fixation on words, their influences do not jointly constrain lexical candidates, resulting in an additive effect. Regarding landing position, we expect that initial fixations on three-character words will land further into the word than those on single-character words, and that fixations on target words in high-constraint contexts will land further into the word than those in low-constraint contexts. However, it remains an open question whether there is an interaction between contextual constraint and word length.

## Method

### Participants

The sample size was determined using the *mixedpower* package in R (Kumle et al., 2021). A power analysis on the initial 20 participants showed that 40 participants would be required to reach 85% power. Accordingly, 48 native Chinese speakers (22 males; age 18–25 years, *M* = 20.46, *SD* = 1.86) were recruited from a university in mainland China. All participants had normal or corrected-to-normal vision and were unaware of the purpose of the experiment.

### Materials

We selected 30 single-character words and 30 three-character words as target words, and matched them on the number of strokes in the first character (*M*_single-character_ = 8.48, *SE* = 0.45; *M*_three-character_ = 7.63, *SE* = 0.42, *t* (58) = −1.39, *p* = 0.15) and word frequency (*M*_single-character_ = 10.10, *SE* = 1.79; *M*_three-character_ = 9.56, *SE* = 2.15, *t* (58) = −0.19, *p* = 0.85) (Cai and Brysbaert, 2010). These target words were embedded in 60 pairs of experimental sentences. Each pair contained an equal number of characters and differed in the first part of the sentences (from the sentence beginning up to the second or third character preceding the target word) to create either a high-constraint or low-constraint context for the target word. Experimental sentences consisted of 15–22 characters (*M* = 19.53, *SD* = 1.59), with at least five characters preceding and following the target word.

**Figure 1:**
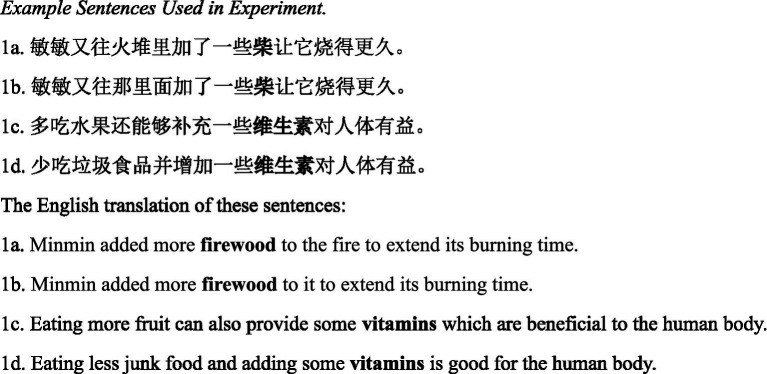
Example sentences used in experiment. In sentence *a* and *b*, the target word was single-character word “柴/firewood,” while in sentence *c* and *d*, the target word was three-character word “维生素/vitamins.” The target words are highly predictable in the *a, c* version sentences and unpredictable in the *b, d* version sentences. The target words are highlighted in bold in the examples for illustrative purposes, but was not used during the actual experiment.

Counterbalancing of sentence frames across the four conditions was accomplished using a Latin square design, resulting in four lists of 60 sentences. Each participant read 15 sentences in each of the four cells defined by the combinations of constraint and word length. An example sentence and its English translation are shown in Figure 1.

#### Rating of materials

In this paper, we operationalize “high/low contextual constraint” as the cloze probability of the target word. To determine the degree of constraint, 60 students who did not participate in the formal experiment were recruited to complete a cloze probability task. They were presented with the first part of each sentence up to (and including) the character to the left of the target word and were asked to provide the next word in the sentence (i.e., predict the target word). A total of 60 sentence pairs were divided into two lists, so that the two sentence contexts containing the same target word did not appear in the same list. Participants were randomly assigned to one of the lists. We classify cloze probability > 0.67 as high- constraint and cloze probability < 0.30 as low-constraint. The average cloze probability of the target word in high/low constraint contexts is *M* = 0.88 (*SD* = 0.10, range: 0.67–1) and *M* = 0.20 (*SD* = 0.06, range: 0.00–0.30), *t* (59) = 50.93, *p* < 0.001.

In addition, a plausibility rating study was conducted to evaluate how well the target words fit into the sentences. 32 participants were presented with the first part of the sentence up to (and including) the target words and were asked to rate the plausibility of the sentence (assuming that the sentence will end with a second part) on a 5-point scale (where 1 = highly implausible; 5 = highly plausible). Two counterbalanced material sets were created, and participants were randomly assigned to one of them. All sentences received high ratings for plausibility (*M* = 4.63, *SD* = 0.19), although high-constraint sentences (*M* = 4.80, *SD* = 0.21) were rated higher than low-constraint sentences (*M* = 4.47, *SD* = 0.36), *t* (59) = 5.92, *p* < 0.001.

### Apparatus and procedure

An SR EyeLink 1000 eye tracker system was used to record participants’ eye movements at a rate of 1,000 Hz. Participants read sentences presented on a 27-inch monitor with a 1,920 × 1,080 pixels resolution and a screen refresh rate of 240 Hz. The experimental materials were presented in black on a gray background. All characters were printed in the Song font and each character subtended approximately 1 degree of visual angle with the participant’s eyes being 57 cm away from the monitor.

The experiment consisted of a calibration phase and an experimental phase. In the calibration phase, each subject performed a 3-point calibration procedure to ensure that the eye-tracker recordings were accurate. The experimental phase then followed. Participants were instructed to read each sentence carefully for comprehension. At the beginning of each trial, a fixation point appeared at the first character of the sentence. Participants were required to fixate on this point continuously before the sentence was displayed. Eight practice sentences were presented at the beginning of the experiment to familiarize participants with the procedure. Each participant read the 60 experimental and 48 filler sentences in a random order, and one-third of the sentences were followed by a true-false comprehension question. The experiment lasted approximately 30 min.

### Data analysis

The mean accuracy on comprehension questions was 97.01% (*SD* = 0.17), indicating that participants understood the sentences well. Fixations shorter than 60 ms or longer than 600 ms were excluded from the analysis (2.85% of the data). Trials were excluded if there was track loss or blink in the pretarget, target, or posttarget regions (1.84% of the data), and trials with more than five blinks during sentence reading were excluded (1.32% of the data). Trials with inaccurate saccade landing at the target word were excluded (4.76% of the data). In addition, to avoid inclusion of extremely long saccades, any saccade launch sites longer than five characters were also excluded (3.67% of the data). Ultimately, 2,378 trials (82.57% of the valid trials) remained, and no significant differences were found across the four conditions (*ps* > 0.064).

We first report eye movement measures for the target word region: *Skipping rate* (SKIP, the probability of skipping the target word in the first reading), *First fixation duration* (FFD, the duration of the first eye fixation on the target word during the first pass through the sentence), *Single fixation duration* (SFD, the duration spent on the initial fixation on the target word given that the reader made only one fixation on the word on the first pass reading), *Gaze duration* (GD, the sum of the duration of all first pass fixations on the target word, before the eyes first leave the target word to either the left or right), and *Second pass reading time* (Second, the sum of all fixations on the target word following the initial first-pass reading). Skipping rate and first-fixation duration reflect the early stage of processing, such as lexical access. Gaze duration is influenced by both lexical access and integration process, while second pass reading time reflects later processing, such as sentence integration or error correction (Rayner, 1998, 2009).

We also analyzed saccade measures: *Launch site* (the distance between the last fixation to the left of the target word region and the left side of the target region), *Saccade length* (the length of the saccade that first enters the target word region), *Landing position* (the distance between the first fixation within the target word and the left side of the target region), and *the proportion of the initial landing positions* in the length-matched region analyses. As demonstrated by Li et al. (2011) and Ma et al. (2019), the regions of interest (ROIs) were length-matched across word-length conditions. For single-character words, the ROI included the target character and the two following characters; for three-character words, it included all three characters. We analyzed the launch site, saccade length, landing position, and the proportion of initial landing positions within these length matched ROIs to examine whether Chinese readers tend to fixate on the center of a given word when contextual information facilitates word segmentation. All measures were recorded in characters, with each character occupying 48 pixels.

Statistical analyses were performed using linear mixed-effects models (LMMs) for reading times, landing position, launch site and saccade length, and generalized linear mixed models (GLMMs) for skipping rate. We used the lme4 package in R (R Development Core Team, 2020). In these models, fixed effects were centered, with factor levels coded as −0.5 and 0.5. The models included two main effects (constraint: high vs. low, and word length: single-character vs. three-character) and their interaction. Random effects were subjects and items, with maximal random effects structure (Barr et al., 2013). If convergence was not achieved, we sequentially removed slopes and/or intercepts for subjects and/or items until the model converged. The *lmerTest* package was used to calculate *p*-values (Kuznetsova et al., 2017).

## Results

The means (and standard errors) of the eye movement measures are presented in Table 1, and the results of the linear mixed model analysis are presented in Table 2.

**Table 1:** Means (and standard errors) of eye-movement dependent measures.

Measure	Single-character		Three-character	
	High	Low	High	Low
SKIP	0.60 (0.02)	0.55 (0.02)	0.12 (0.01)	0.10 (0.01)
FFD	235 (3.2)	248 (3.4)	210 (2.7)	227 (3.1)
SFD	235 (3.2)	248 (3.4)	208 (2.7)	225 (3.1)
GD	243 (3.6)	253 (3.7)	253 (5.0)	276 (5.3)
Second	237 (5.4)	240 (5.3)	276 (7.4)	289 (8.0)
Launch site	1.85 (0.05)	1.85 (0.05)	1.89 (0.05)	1.87 (0.04)
Saccade length	3.13 (0.05)	3.07 (0.05)	3.41 (0.05)	3.31 (0.05)
Landing position	1.24 (0.03)	1.12 (0.03)	1.38 (0.03)	1.29 (0.03)

Times were measured in milliseconds. Launch site, saccade length and landing position were measured by characters.

**Table 2:** Statistical results from the linear mixed model of the eye-movement dependent measures.

Measure	Contrast	*b*	*SE*	*t/z*	*p*
SKIP	CC	−0.25	0.11	**−2.16**	**0.031**
	WL	−2.71	0.15	**−18.37**	**<0.001**
	CC × WL	−0.01	0.23	−0.06	0.96
FFD	CC	14.27	3.68	**3.88**	**<0.001**
	WL	−22.38	5.21	**−4.30**	**<0.001**
	CC × WL	6.51	7.35	0.89	0.38
SFD	CC	14.78	3.91	**3.78**	**<0.001**
	WL	−23.94	5.30	**−4.52**	**<0.001**
	CC × WL	7.04	7.79	0.90	0.37
GD	CC	17.50	5.77	**3.03**	**0.002**
	WL	22.24	8.78	**2.53**	**0.014**
	CC × WL	15.62	11.52	1.36	0.18
Second	CC	7.88	13.24	0.60	0.55
	WL	42.88	14.04	**3.06**	**0.003**
	CC × WL	2.05	26.49	0.08	0.94
Launch site	CC	−0.004	0.04	−0.09	0.93
	WL	0.03	0.08	0.38	0.70
	CC × WL	−0.007	0.09	−0.08	0.93
Saccade length	CC	−0.08	0.04	**−1.84**	**0.066**
	WL	0.28	0.08	**3.44**	**0.001**
	CC × WL	−0.02	0.08	−0.28	0.78
Landing position	CC	−0.11	0.03	**−3.66**	**<0.001**
	WL	0.16	0.04	**3.68**	**<0.001**
	CC × WL	0.03	0.06	0.42	0.68

CC = the contextual constraint effect; WL = the word length effect. Model specification is described in the text. Significant |*t*| or |*z*| values are in bold.

### Skipping rate

There was no interaction between contextual constraint and word length on skipping the target word (*b* = −0.01, *SE* = 0.23, *z =* −0.06, *p* = 0.96). The main effects of contextual constraint and word length were significant. The skipping rate on the target word was lower in the low-constraint context (*M* = 0.32, *SE* = 0.01) than in the high-constraint context (*M* = 0.36, *SE* = 0.01; *b* = −0.25, *SE* = 0.11, *z* = −2.16, *p* = 0.031), and lower for three-character targets (*M* = 0.11, *SE* = 0.01) than for single-character targets (*M* = 0.57, *SE* = 0.02; *b* = −2.71, *SE* = 0.15, *z* = −18.37, *p* < 0.001).

### Fixation times

No significant interaction was found between contextual constraint and word length for all fixation times on the target word (*ts* < 1.36, *ps* >0.18). The main effect of contextual constraint was significant on all early reading time measures (FFD: *b* = 14.27, *SE* = 3.68, *t* = 3.88, *p* < 0.001; SFD: *b* = 14.78, *SE* = 3.91, *t* = 3.78, *p* < 0.001; GD: *b* = 17.50, *SE* = 5.77, *t* = 3.03, *p* = 0.002), as readers spent more time on target words in the low-constraint context than in the high-constraint context. The main effect of word length was significant on all reading time measures. Specifically, the early fixation times on target words were shorter for three-character than for single-character targets (FFD: *b* = −22.38, *SE* = 5.21, *t* = −4.30, *p* < 0.001; SFD: *b* = −23.94, *SE* = 5.30, *t* = −4.52, *p* < 0.001), while gaze duration and second pass reading time were longer for three-character than for single-character targets (GD: *b* = 22.24, *SE* = 8.78, *t* = 2.53, *p* = 0.014; Second: *b* = 42.88, *SE* = 14.04, *t* = 3.06, *p* = 0.003).

### Launch site

There were no significant effects across conditions on launch site into the target word region (*ts* < 0.38, *ps* > 0.70).

### Saccade length

There was no interaction between contextual constraint and word length for the length of the saccade into the target word (*b* = −0.02, *SE* = 0.08, *t* = −0.28, *p* = 0.78). The main effect of contextual constraint was marginally significant, as saccade lengths into the target word in the low-constraint context (*M* = 3.19, *SE* = 0.05) were shorter than those in the high-constraint context (*M* = 3.27, *SE* = 0.05; *b* = −0.08, *SE* = 0.04, *t* = −1.84, *p* = 0.066). The main effect of word length was significant, as saccade lengths into three-character targets (*M* = 3.36, *SE* = 0.05) were longer than those into single-character targets (*M* = 3.10, *SE* = 0.05; *b* = 0.28, *SE* = 0.08, *t* = 3.44, *p* = 0.001).

### Landing position

No interaction between contextual constraint and word length was observed for landing positions in the target word (*b* = 0.03, *SE* = 0.06, *t* = 0.42, *p* = 0.68). The main effects of contextual constraint and word length were significant. The initial landing positions were closer to the beginning of target words in the low-constraint contexts (*M* = 1.23, *SE* = 0.03) than in the high-constraint contexts (*M* = 1.31, *SE* = 0.03; *b* = −0.11, *SE* = 0.03, *t* = −3.66, *p* < 0.001), and were located further into three-character targets (*M* = 1.34, *SE* = 0.03) than into single-character targets (*M* = 1.18, *SE* = 0.03; *b* = 0.16, *SE* = 0.04, *t* = 3.68, *p* < 0.001).

### The proportion of the initial landing positions

We computed the proportion of initial landing positions in the three-character ROIs under different conditions, and found no significant difference between three-character words and single-character words across high and low-constraint contexts (*p* = 0.82).

### Bayes factor analysis

In addition, to determine the strength of evidence for the null interactive effect between contextual constraint and word length across all measures, we computed Bayes factors using the *lmBF()* function from the BayesFactor package in the R environment (Morey et al., 2015). Specifically, we calculated Bayes factors separately for the interactive and additive effects of contextual constraint and word length on all measures, to assess support for H_1_ over H_0_ (i.e., BF_10_; see Morey and Rouder, 2011). Bayes factors (BFs) > 10 were interpreted as indicating strong support for the model including the interaction effect (H₁), while BF > 3 provided moderate support for H₁, BF ≈ 1 suggested no evidence in favor of H₁, and BF < 1/3 indicated moderate evidence in favor of the additive model (H_0_).

The results showed that, compared with the additive model, the BF_10_ for all dependent measures in the interaction model were less than 1/3 (SKIP: BF_10_ = 0.10; FFD: BF_10_ = 0.12; SFD: BF_10_ = 0.14; GD: BF_10_ = 0.23; Second: BF_10_ = 0.13; Launch site: BF_10_ = 0.06; Saccade length: BF_10_ = 0.07; Landing position: BF_10_ = 0.07), indicating strong evidence in favor of H₀, that is, contextual constraint and word length do not interact to influence eye-movement behavior.

## Discussion

This study examined whether contextual constraint modulated the processing of word length information during Chinese reading. The results revealed significant main effects of contextual constraint and word length on skipping rates, fixation times, saccade lengths, and landing positions. The distribution of initial landing positions did not show significant differences between the three-character and single-character conditions, regardless of whether the contextual constraint were high or low. Moreover, Bayes factor analyses provided strong evidence that there was no interaction between contextual constraint and word length across any measures. These findings suggest that contextual constraint and word length do not interact in influencing eye movement control, and that Chinese readers did not rely on word length information to guide initial landing positions toward the word center, even when contextual cues were available.

These results align with findings in English reading, which report independent effects of contextual constraint and word length on fixation durations and word skipping (Drieghe et al., 2004; Rayner et al., 2011). The fact that a similar pattern emerged in Chinese, despite the absence of interword spaces, suggests a universal mechanism underlying word processing across languages (Li et al., 2014; Li X. et al., 2022; Liversedge et al., 2016, 2024). Nevertheless, previous research has demonstrated that sentence context facilitates word segmentation in Chinese reading (Huang and Li, 2020; Huang et al., 2021). Given that word segmentation is closely related to word length recognition, the lack of an observed interaction between contextual constraint and word length requires further discussion.

Why does contextual constraint fail to modulate the processing of word length information? One possible explanation is that context and word length operate at distinct stages of lexical processing. The current study showed that contextual constraint influenced early eye movement measures (skipping rate, first fixation duration, and landing position), but had no effect on second pass reading time, which reflects post-lexical integration (Kliegl et al., 2006; Staub, 2015). In contrast, word length effects were significant at both early and late stages: three-character words had farther landing positions, lower skipping rates, and longer gaze durations and second pass times on the target words than single-character words. The shorter early fixation times on three-character words may reflect more efficient initial lexical access due to their higher informational density or morphological complexity. In contrast, the longer gaze durations and second-pass times likely indicate increased integration effort or semantic processing load associated with longer words, consistent with previous findings (Ma et al., 2019; Zang et al., 2018). These findings are consistent with the E-Z Reader model (Reichle et al., 1998, 2003), which posits that contextual constraint primarily influences L1, while word length affects both the L1 and L2 stages. Because these factors act at different stages, their effects are additive rather than interactive.

Another plausible explanation involves the availability of parafoveal lexical information. During natural reading, readers extract information not only from the currently fixated word (foveal vision) but also from upcoming words (parafoveal vision). It is well established that English readers can access word length information from parafoveal vision. Studies have shown that parafoveal word length information modulates the effects of contextual constraint: when parafoveal word length was accurate, contextual effects on the target word were significant; when it was inaccurate, contextual effects disappeared (Juhasz et al., 2008; White et al., 2005). Although Chinese lacks explicit interword spacing, readers can still perceive word boundaries (Yang et al., 2012). Previous research has also shown that parafoveal processing plays a crucial role in saccade target selection in Chinese reading (Li et al., 2015; Liu et al., 2015, 2016). This points to a strong bottom-up mechanism, where stable parafoveal cues may override top-down contextual expectations. Future studies should further investigate the interplay between parafoveal word length cues and contextual information in Chinese reading.

**Figure 2:**
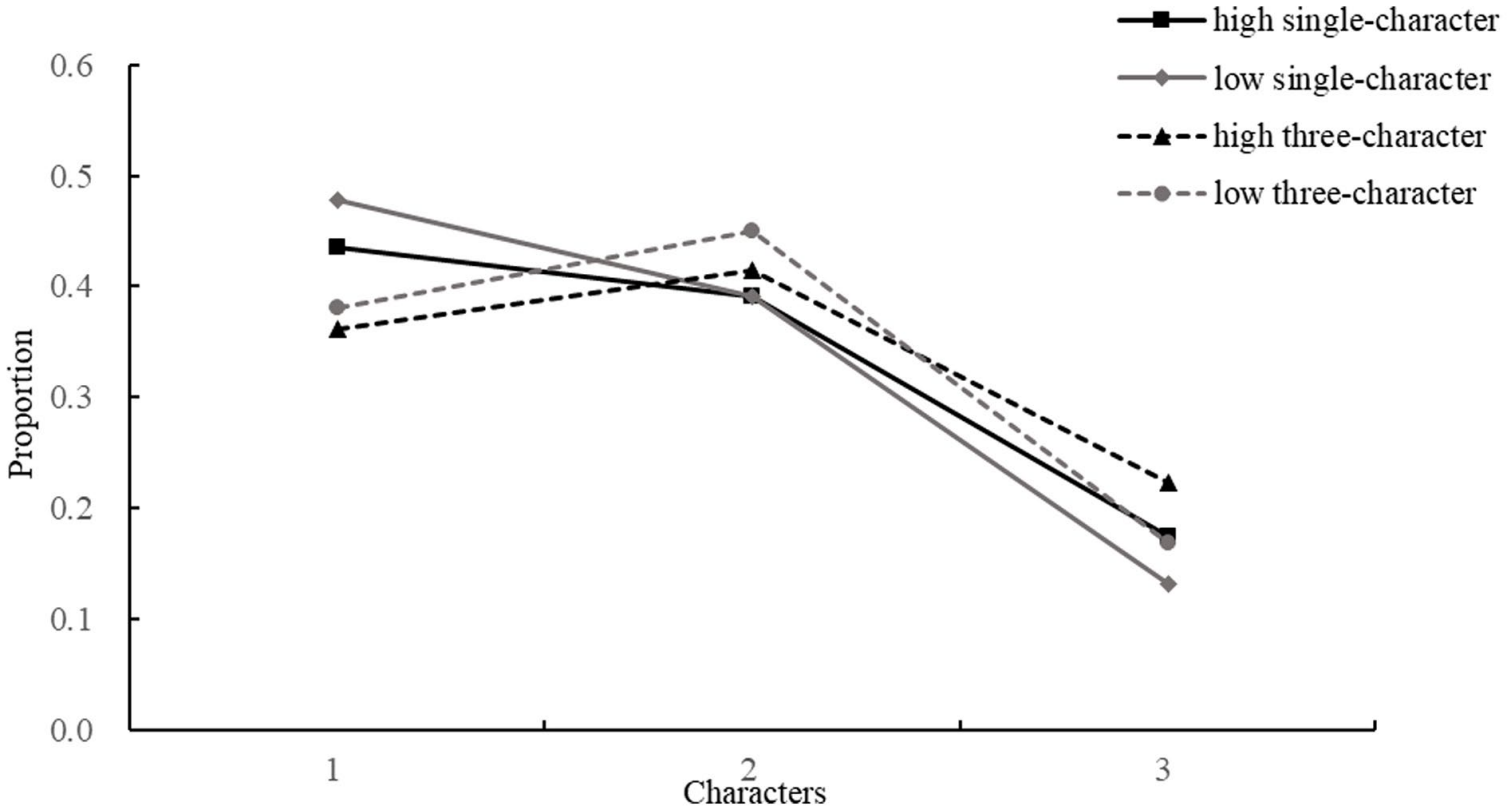
The proportion of the initial landing positions in the three-character ROIs across different conditions.

Additionally, the study did not find that contextual constraint facilitated the initial landing position at the center of a word. First, we did not find any interaction between contextual constraint and word length on saccade lengths or landing positions within the length-matched region, indicating that contextual constraint and word length do not jointly modulate saccade target selection. Second, initial landing positions were more likely at the beginning of the three-character ROIs in the single-character condition but near the middle in the three-character condition. However, we did not find significant differences in the proportion of initial landing positions across different levels of contextual constraint and word length (see Figure 2). Consistent with earlier studies (Li and Shen, 2013; Zang et al., 2013), the current findings suggest that under naturalistic reading conditions, readers do not employ word-based saccade targeting, even when contextual cues facilitate word segmentation. Although our findings do not support word-based saccade targeting, future research could explore whether Chinese readers rely on combination of character-based and word-based (Li et al., 2011), or processing-based (Wei et al., 2013) and dynamic-adjustment (Liu et al., 2015, 2016) strategies for saccade targeting, particularly in the absence of clear word boundaries.

The results of this study have important implications for existing models of reading. First, although the classic E-Z Reader model can successfully reproduce the eye movement patterns in English reading, it does not incorporate a word segmentation module, making it difficult to capture the impact of context on word segmentation in the continuous script of Chinese. Second, although the existing CRM is based on the interactive activation framework (McClelland and Rumelhart, 1981), it has not yet modeled how higher-level context regulates lower-level visual lexical processing. The findings of this study suggest that contextual pre-activation and word length cues may drive eye movement behavior through parallel and relatively independent pathways during reading. Therefore, it is necessary to introduce a “context activation subsystem” and a “word length cue subsystem” into the model architecture and to allow the two to exert a weighted combined effect on fixation time and saccades. These results prompt improvements to the existing theoretical model: on the one hand, a top-down contextual adjustment connection should be added to the CRM to achieve a dynamic influence on the recognition of word segmentation boundaries; on the other hand, the bottom-up transmission pathway of word length cues should be retained to simulate the real-time decoding of visual-morphological information. Finally, this study not only provides new empirical evidence for theoretical models of reading but also lays the foundation for subsequent exploration of the neural mechanisms underlying the integration of contextual and lexical information. Future work could combine electroencephalography (EEG) or the coregistration of eye movements with EEG measurements to further reveal the spatiotemporal characteristics of these two processing pathways in reading.

In summary, this study investigated whether contextual constraint modulates the processing of word length information during Chinese reading. The findings revealed that both contextual constraint and word length influenced eye-movement behavior. However, no evidence supported an interaction between these factors. Furthermore, we did not find reliable evidence that Chinese readers preferentially fixate the word center based on word length, regardless of contextual constraint. These results provide direct empirical evidence that contextual constraint did not modulate the processing of word length information in Chinese reading and offer a basis for improving cognitive models of eye-movement control in Chinese reading.

## Data Availability

The datasets presented in this study can be found in online repositories. The names of the repository/repositories and accession number(s) can be found in the article/supplementary material.
